# D-chiro-inositol effectively counteracts endometriosis in a mouse model

**DOI:** 10.1186/s10020-025-01178-6

**Published:** 2025-04-11

**Authors:** Martina Placidi, Giovanni Casoli, Teresa Vergara, Andrea Bianchi, Domenica Cocciolone, Silvia Zaccardi, Guido Macchiarelli, Maria Grazia Palmerini, Carla Tatone, Arturo Bevilacqua, Giovanna Di Emidio

**Affiliations:** 1 Department of Life, Health and Environmental Sciences, University of L’Aquila, Via G. Petrini, 67100 L’Aquila, Italy; 2 Department of Dynamic, Clinical Psychology and Health Studies, Sapienza University of Rome, 00185 Rome, Italy; 3 Research Center in Neurobiology Daniel Bovet (CRiN), Systems Biology Group Lab, Rome, Italy; 4 The Experts Group on Inositol in Basic and Clinical Research and on PCOS (EGOI-PCOS), 00156 Rome, Italy

**Keywords:** DCI, DG, Endometriosis, Aromatase, EMT, Sirt1, E-Cadherin

## Abstract

**Background:**

Endometriosis, a common condition affecting 5–10% of women of reproductive age, is the growth of endometrial-like tissue outside the uterus, leading to pain and infertility. Current treatments, such as surgery and hormonal therapy, offer limited long-term benefits. This study investigated the potential of D-chiro inositol (DCI), a natural compound that influences ovarian steroidogenesis, to treat endometriosis and compared its efficacy with a progestin drug such as Dienogest (DG).

**Methods:**

We established a non-surgical mouse model of endometriosis in CD1 mice. Uterine horns were removed from donor mice, cut into fragments and inoculated in recipient mice by intraperitoneal injection. Endometriosis progression was assessed at 15, 21 and 28 days after transplantation, with the 28-day window being the most effective. The mice were then randomly assigned to four experimental groups, which received for 28 days: water (EMS); DCI 0.4 mg/die (DCI); DCI 0.2 mg/die and Dienogest 0.33 ng/die (DCI + DG); DG 0.67 ng/die (DG). At the end of the treatments, endometriotic lesions, ovaries and circulating estradiol levels were analyzed.

**Results:**

The results showed that treatment with DCI, both alone and in combination with DG, significantly reduced the number, size and vascularization of endometriotic lesions compared to the EMS control group. Histological analysis confirmed a decrease in endometriotic foci across all treatment groups, with the most pronounced effects in the DCI group. To investigate the underlying molecular mechanisms, we found that DCI led to a significant reduction in the expression of Sirt1 and an increase in E-Cadherin, indicating a reduction in EMT transition relevant for lesion development. In addition, DCI decreased cell proliferation and,blood vessel formation, as evaluated by PCNA and CD34, respectively. Futhermore, in the ovary, DCI treatment downregulated the expression of aromatase (Cyp19a1), the enzyme critical for estrogen biosynthesis, and increased the number of primordial to antral follicles, suggesting a beneficial effect on ovarian folliculogenesis.

**Conclusions:**

By modulating proliferation, EMT transition and aromatase activity, DCI emerges as a promising compound for endometriosis treatment.

**Graphical Abstract:**

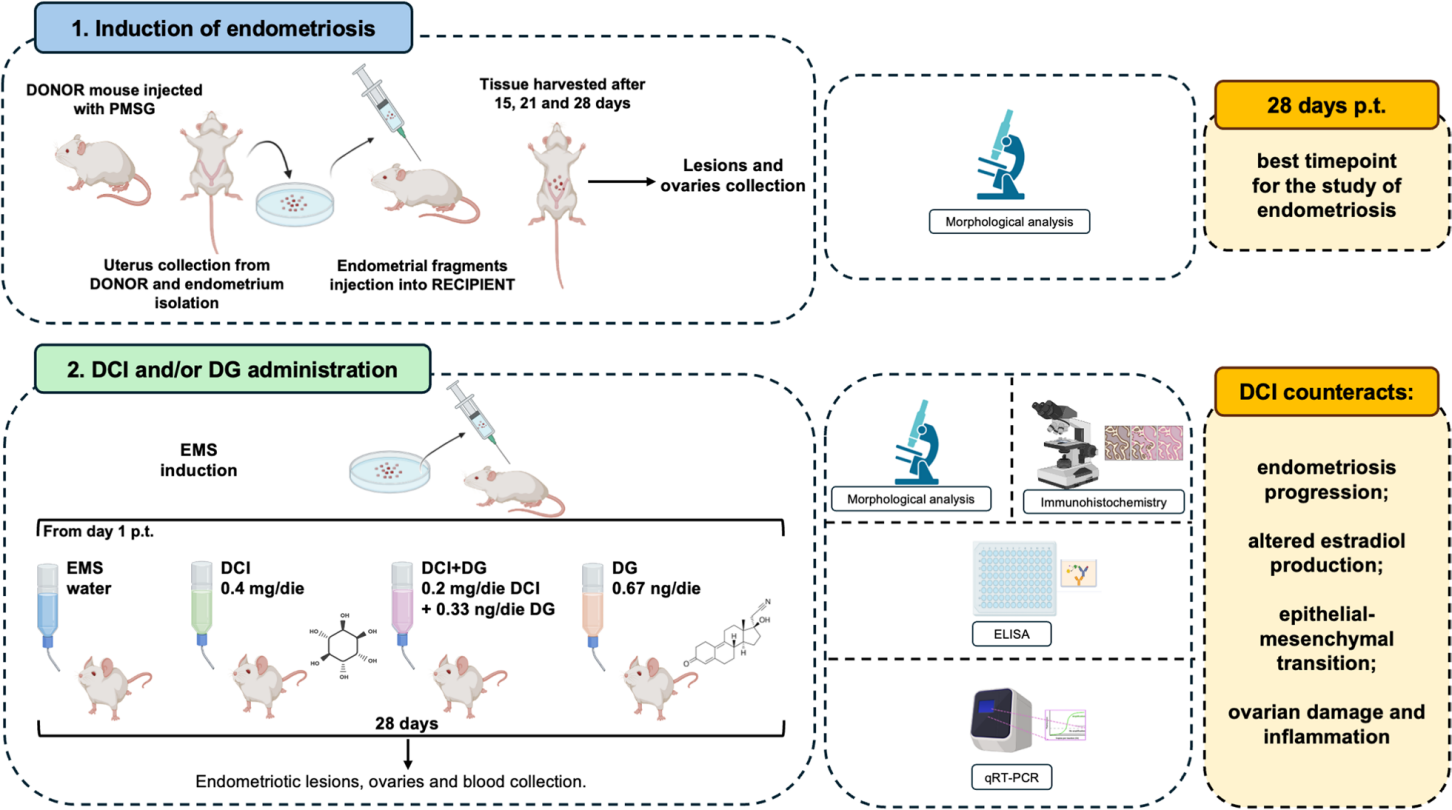

**Supplementary Information:**

The online version contains supplementary material available at 10.1186/s10020-025-01178-6.

## Introduction

Endometriosis is a gynecologic disorder with heterogeneous pathologic features, affecting 5–10% of fertile women of reproductive age and up to 40% of infertile women (Carson and Kallen 2021). It is characterized by the presence of endometrial-like tissue containing both glands and stroma outside the uterine cavity, forming ectopic endometriotic lesions that cause dyspareunia, chronic pelvic pain, dysmenorrhea, and infertility (Sampson 1927). Although the causes of this pathology are still debated, the phenomenon of retrograde menstruation is the most valuable theory. The disease reduces the quality of life of affected women and is associated with non-negligible health care costs (Dydyk and Gupta 2024). In addition, the occurrence of endometriotic lesions has been associated with the development of ovarian cancer if left untreated (Brinton et al. 1992).

Factors associated with the disease include an alteration of the immune response (Lebovic et al. 2001), stimulation of endometrial cell proliferation (Surrey and Halme 1990) and the ability of endometrial cells to go undergo the epithelial-mesenchymal transition (Yang and Yang 2017), degrade the surrounding extracellular matrix, adhere to and invade the peritoneal surface through the production of adhesion molecules and matrix metalloproteinases (MMPs) (Sillem et al. 1998; Prifti et al. 1998; Chung et al. 2001). MMPs induce the release of ECM-bound growth factors and cytokines (Mott and Werb 2004; Kalu et al. 2007; Khan et al. 2008, 2009) leading to a significant degree of peritoneal inflammation, angiogenesis, and tissue remodeling.

Peritoneal inflammation may represent a cause of infertility possibly affecting fallopian tube-ovarian function and/or causing alterations in gamete transport, and a peculiar dependence on circulating estrogens and resistance to progesterone (Xu et al. 2012). In addition besides inflammation, angiogenesis within the lesions is an additional factor in disease progression, as endometriotic tissue develops the ability to promote vascularization within and around the tissue, allowing it to become self-sustaining (Nisolle et al. 1993; Fujimoto et al. 1999). Interestingly, Vascular Endothelial Growth Factor (VEGF), the most potent and specific angiogenic factor, is increased in the peritoneal fluid of women with endometriosis, along with higher macrophage counts, under apparent regulation by ovarian steroids (McLaren et al. 1996; Shifren et al. 1996; Donnez et al. 1998; McLaren 2000). Clinical treatment of endometriosis focuses on the relief of symptoms such as pain and management of subfertility and includes pharmacologic and surgical interventions.

The disease exhibits a peculiar dependence on circulating estrogens and can develop resistance to progesterone (Xu et al. 2012). The observation that symptoms of endometriosis usually improve during pregnancy (Leeners et al. 2018) has led to a therapeutic approach aimed at reducing circulating estrogen to post-menstrual or postmenopausal levels. These treatments focus on reducing ovarian estrogen secretion with gonadotropin-releasing hormone (GnRH) agonists or antagonists (Brown and Farquhar 2014) and include oral contraceptives, androgens, and aromatase inhibitors. Although the mechanisms of action of these medications are still unclear, the European Society of Human Reproduction and Embryology (ESHRE) guidelines for the treatment of patients with endometriosis recommend the concomitant use of inhibitors of the enzyme aromatase, which is responsible for the conversion of androgens into estrogens, and oral contraceptives, progestins, or GnRH agonists (Becker et al. 2022).

However, hormonal treatments have limited benefits and cause adverse effects that include hot flashes, weight gain, and mood disorders (Hughes et al. 2007), as well as an overall reduction in the quality of life (Bergqvist 1999). Since it is known that a reduction in estrogen levels promotes osteoporosis and increases the risk of heart disease, therapy for endometriosis is recommended for a maximum of 6 months. Thus, the main alternative to hormones currently recognized to solve this problem is surgical intervention to remove the lesions (Becker et al. 2022), making the search for new therapeutic approaches urgent.

Based on several recent observations from our and other research groups, D-chiro-inositol (DCI) may represent a candidate as a novel therapeutic approach to endometriosis. D-chiro-inositol is a natural molecule that belongs to the family of inositols, essential components of plasma membrane phospholipids that are detectable in every cell of the body. DCI and its more abundant isomer myo-inositol (MI) are constituents of phosphoglycans that act as second messengers of insulin (Low and Saltiel 1988; Larner 2002; Bevilacqua and Bizzarri 2016). Indeed, the use of a specific combination of these molecules, with a 40:1 molar ratio of MI to DCI ratio, has been shown to improve spontaneous fertility or the success rate of assisted reproduction techniques (ARTs) (Colazingari et al. 2013) as well as a reliable treatment for insulin resistance (Facchinetti et al. 2015) in women with polycystic ovary syndrome (PCOS).

In previous work using mouse models of PCOS (Bevilacqua et al. 2019, 2021), we confirmed the efficacy of the 40:1 combination of MI and DCI in rapidly restoring the normal ovarian histology and fertility. However, at the same time, we found that formulations containing high doses of DCI had an opposite effect, exacerbating the PCOS-like ovarian features and prolonging the infertile status of the mice over time. High daily doses of DCI, equivalent to the human formulations 1200 mg normally used to treat PCOS, altered ovarian histology, increased serum testosterone levels and decreased the expression of ovarian aromatase. This observation represents the first evidence of a specific DCI-mediated downregulation of aromatase expression in an in vivo system and supports a similar result obtained in cultured human granulosa cells (Sacchi et al. 2016). These findings provide the basis for our hypothesis that treatments with DCI would have clinical applications in pathological conditions, such as endometriosis, that require reduced estrogen levels. Clinical evidence on DCI already supports its use in estrogen-dependent pathologies, such as endometrial hyperplasia (Porcaro et al. 2023). Furthermore, as a natural molecule, DCI lacks the adverse effects typical of direct hormonal treatments or the use of enzyme inhibiting chemical compounds, such as letrozole. Finally, the available oral formulations show good bioavailability, safe pharmacologic profiles and beneficial effects as observed on insulin-resistant patients.

To investigate the possibility of using DCI in the treatment of endometriosis, we have designed an experimental protocol at the preclinical level in a mouse model. Such a model was established by transplantation of endometrial fragments into the peritoneal cavity of recipient female mice, as previously described (Dabrosin et al. 2002; Xu et al. 2012), with some modifications. The advantages of this model include the absence of rejection, which allows long-term studies, and the possibility to study: (a) the adhesion mechanisms of the ectopic endometriotic tissue; (b) the immunological aspects of the pathology; (c) the adverse effects of endometriosis on ovarian histological and physiological features; and (d) the efficacy of a potential therapeutic treatment at the morphometric, histological, cellular and molecular levels. Overall, our preclinical evaluations showed that DCI reduces pathological features of both endometriotic lesions and ovarian tissue and can be considered a “novel candidate” molecule for the treatment of endometriosis.

## Materials and methods

### Animals

Outbred CD-1 mice (Charles River Laboratories Italia s.r.l., Calco, Italy) were maintained in a temperature-controlled environment under a 12 h light/dark cycle (07:00–19.00) with ad libitum access to food and water.

All experiments were carried out in conformity with national and international laws and guidelines (National Institutes of Health Guide for the Care and Use of Laboratory Animals, NIH publication no. 85–23, 1985; European Economic Community Council Directive 86/609, OJ 358, 18 December 1986; Italian Legislative Decree 116/92, Gazzetta Ufficiale della Repubblica Italiana n. 40, 18 February 1992) and were specifically approved by the Internal Committee of the University of L’Aquila and the Italian Ministry of Health (authorization no. 917/2023-PR). Mice were sacrificed by inhalant overdose of carbon dioxide (CO_2_, 10–30%), followed by cervical dislocation. All efforts were made to minimize suffering. Details regarding the experimental procotol are summarized in Supplementary Table 1 and 2 according to the ARRIVE guidelines 2.0 (Percie du Sert et al. 2020).

### Induction of endometriosis

To induce endometriosis, we used a syngeneic mouse model using naturally cycling mice as described by Richards et al. (Richards et al. 2020). In preliminary experiments, four female mice at the diestrus stage of the ovulatory cycle, as confirmed by vaginal smears, were selected as tissue donors and primed with a single intraperitoneal (i.p.) injection of 5 IU of pregnant mare serum gonadotropin (PMSG, Folligon Intervet-International, Boxmeer, The Netherlands) to induce folliculogenesis and endometrial cell proliferation. Mice were sacrificed 46 h later; the uteri were rapidly excised under sterile conditions and placed in a Petri dish containing phosphate buffered saline (PBS). Under a dissecting stereomicroscope, the uterine horns were separated and opened longitudinally with a scalpel blade. The decidualized endometrium was dissected from the outer capsule (serosa and myometrium) and then fragmented into pieces of approximately equal size, 0.5 mm thick. Endometrial fragments were transferred to sterile PBS for inoculation into the peritoneal cavity of recipient mice, previously anesthetized by i.p. injection of 25 mg/ml ketamine and 5 mg/ml xylazine. Equal numbers of endometrial fragments in 0.4 ml PBS were loaded into a 1 ml syringe and transplanted i.p. into recipient mice using an 18-gauge needle. To minimize variability due to differences in endometrial tissue characteristics among donor mice, all collected endometrial tissue fragments were pooled prior to transplantation, ensuring that each recipient mouse received a homogenous mixture rather than tissue from a single donor. The ratio of donor to recipient mice was 1:4. After transplantation, mice were returned to normal housing conditions and sacrificed after 15, 21 and 28 days, corresponding to approximately 4, 5 and 7 ovulatory cycles respectively (3.7, 4.6, and 6.5 months in humans), to assess the progression of endometriosis.

At 15, 21, 28 days post-transplantation (p.t.), recipient mice were sacrificed. The intestinal cavity of each mouse was exposed for visual inspection and counting of endometriotic lesions under a dissecting stereomicroscope. The lesions were then excised and transferred to a petri dish, measured along two axes through an eyepiece equipped with a micrometric scale (Ahmed et al. 2021) and examined for presence of blood vessels.

Since the pathological development of endometriotic lesions appeared to be time-dependent, with the size, number, vascularization and proliferation of lesions increasing over time, we selected 28 days p.t. as the best time point for further experiments.

### Effects of D-chiro-inositol in mouse endometriosis model

Endometrial fragments were harvested from 7 donor mice and transplanted i.p. into 28 recipient mice as previously described. On day 1 p.t., mice were randomly assigned to four experimental groups. One group received normal drinking water (endometriosis control group, EMS). The other groups received drinking water containing: 0.4 mg/2 ml D-chiro-inositol (DCI); 0.67 ng/2 ml Dienogest (DG), the progestin drug for endometriosis used as a positive control; and 0.2 mg D-chiro-inositol and 0.33 ng DG (DCI + DG). The dose of dienogest was selected based on the commercially available formulations commonly used in women with endometriosis (2 mg/day). For DCI, the dose was based on the 1200 mg/die regimen, which has been safely administered in women with PCOS and is known to reduce androgen levels by modulating aromatase activity (Nestler et al. 1999; Bevilacqua et al. 2021). These treatments were calculated based on previous observations of a mouse’s daily water consumption, approximately 2 ml/day for 20 g of body weight (Bevilacqua et al. 2019), and a previously tested mice/humans’ dose-conversion formula (Nair and Jacob 2016; Bevilacqua et al. 2021). Mice were housed as previously described with bottle replacements every 2–3 days, and weighed weekly during the treatment.

### Specimen collection and preliminary processing

Ovaries were collected from each mouse. One ovary was processed for histological analysis, one was snap frozen in liquid nitrogen and stored at −80 °C until use for molecular analysis.

Part of the lesions were fixed for histological analysis; the remaining lesions were snap frozen in liquid nitrogen and stored at −80 °C until processing for molecular analysis.

To evaluate the effects of D-chiro-inositol in the progression of endometriosis, the blood was immediately collected from the heart after sacrifice using an insulin needle, transferred to an Eppendorf tube containing sodium citrate (100 µl per 1 ml of blood), and centrifuged at 5000 rpm for 5 min. The supernatant was collected, transferred to a new Eppendorf tube, and frozen at −80 °C.

### Haematoxylin and eosin (H&E) staining and Heidenhain’s AZAN trichrome staining

Endometriotic lesions and ovaries were fixed in 3.7% paraformaldehyde (PFA) in PBS (Bio-Optica, Milan, Italy) for 12–16 h dehydrated in ascending graded alcohols, cleared in xylene and embedded in paraffin blocks. Samples were sectioned on a microtome (Leica SMR2000, Wetzlar, Germany) and cut into 6 µm serial sections. Sections were then deparaffinized and rehydrated through a graded series of xylenes and alcohols, stained with H&E according to the manufacturer’s instruction (Bio Optica, Milan, Italy) and observed by light microscopy (LM, Zeiss Axiostar Plus, Oberkochen, Germany).

For Heidenhain’s AZAN Trichrome Staining, paraffin embedded sections of formalin-fixed endometriotic lesions and ovarian tissue were deparaffinized and hydrated through xylenes and graded alcohol series and processed for trichrome staining (Electron Microscopy Sciences, Danvers, MA, USA), according to the manufacturer’s instructions.

### Immunohistochemical analysis

Paraffin-embedded sections of formalin-fixed endometriotic lesions and ovarian tissue were deparaffinized and hydrated through xylenes and graded alcohol series. To enhance antigen retrieval, the sections were boiled in 10 mM citrate buffer (pH 6.1, Bio-Optica, Milan, Italy) in a microwave oven at 720 W (3 cycles/3 min each). The sections were then subjected to a treatment to block endogenous peroxidase activity (Dako). The sections were then incubated with rabbit polyclonal anti-CD34 (1:200, PA1334, Boster Biological technology Pleasanton, CA, USA) (Egorova et al. 2023), anti-IL-1β (1:500, NBP1-42767, Novus biologicals, CO, USA, anti-E-Cadherin (1:200, BS1098, Bioworld Technology, MN, USA) (Ntzeros et al. 2025) antibodies in 1% BSA/PBS overnight at + 4 °C.

For PCNA staining, after thorough washing, sections were incubated with M.O.M mouse IgG blocking reagent (Vector Laboratories Burlingame, CA, USA) overnight at 4 °C according to the manufacturer’s protocol and then incubated with mouse monoclonal anti-PCNA antibody (1:500, NB500-106, Novus Biological) (Mishra et al. 2020) diluted in mouse on mouse (M.O.M) diluent for 30 min, according to the Vector Laboratories instructions. The sections were then washed and incubated with MACH 4 Probe UP534 (Biocare Medical, Pacheco, CA, USA) for 10 min at RT.

Immunocomplexes were detected by MACH1 Universal HRP-Polymer detection (MRH538G, Biocare Medical) followed by 3,3-diaminobenzidine (DAB) and DAB substrate buffer, according to manufacturer’s instructions. Counterstaining was performed with hematoxylin (Bio-Optica). Negative controls were performed by omitting the primary antibody. Finally, the sections were dehydrated and mounted with Neomount (Merck, Darmstadt, Germany). They were observed and photographed under a Leitz Laborlux S microscope (Oberkochen, Germany) equipped with an Olympus digital compact camera.

### Analysis of circulating estradiol levels

Plasma estradiol concentrations were measured by ELISA using the Mouse Estradiol (E2) ELISA KIT CUSABIOR (cat. CSB-E05109m, Wuhan Hi-tech Medical Devices Park, Wuhan, Hubei province, P.R. China), according to the manufacturer’s instructions and a Multiskan^™^ GO microplate spectrophotometer (Thermo Fisher Scientific, Inc., Rockford, IL) set to 450 nm. Experiments were performed in triplicate.

### RNA extraction and real-time reverse transcriptase-polymerase chain reaction analysis

Total RNA from lesions and ovaries was extracted using the GeneAll Ribospin™ 50p kit (cat. KIT304-150, GeneAll Biotechnology Co. Ltd, 05729, Seoul, Korea), according to the manufacturer's instructions. The total RNA concentration and purity were estimated by measuring UV absorbance at 260/280/320 nm using a Lambda25 spectrophotometer (PerkinElmer Inc., Waltham, MA, USA). The resulting RNA (1 µg) was used to obtain cDNA via reverse transcription. The extracted RNA was converted into complementary DNA (cDNA) using a reverse transcription kit (OriGene Technologies, cat. NP100042, Rockville, MD, USA). The cDNA was used for RT-PCR reactions, using the Applied Biosystems 7300 system (Thermo Fisher Scientific, Inc., Rockford, IL) and the TaqMan^®^ Gene Expression Master Mix (Applied Biosystems cat. 4444557) and TaqMan gene expression assays-FAM-MGB according to the manufacturer's instructions. The assay IDs used were 18S-Mm03928990_g1, SIRT1-Mm00490758_m1, and Cyp19a1-Mm00484049_m1. Amplifications were performed as follows: 2 min at 50 °C and 20 s at 95 °C for the initial phase, then 40 cycles of 1 s at 95 °C and 20 s at 60 °C. Gene expression was calculated using the ΔΔCt method (Livak and Schmittgen 2001), using 18S rRNA as reference mRNA and one sample from EMS group as the calibrator.

### Ovarian follicle classification and counting

Follicle classification and counting was performed on at least three serial sections per slide (20 slices in average for ovary), at ~ 50 µm intervals each. Stained ovarian follicles were classified as normal or degenerated for qualitative evaluation. Normal follicles were defined as those with intact basal membrane, absence of pyknotic bodies in the oocyte nucleus, no signs of oocyte and/or granular degeneration. Normal follicles were classified according to Gougeon’s classification (Gougeon 1996) into: (i) primordial follicle, oocyte surrounded by a single layer of flattened pre-granulosa cells; (ii) primary follicle, oocyte showing a single layer of cuboidal granulosa cells; (iii) secondary follicle, with at least two complete layers of granulosa cells; and (iv) antral follicle, with development of an antral cavity.

### Statistical analysis

All experiments were repeated at least three times. All data are presented as mean ± SD of at least three replicates. Normality was assessed by Shapiro–Wilk test. Samples with normal distribution were compared using One-way ANOVA, followed by Tukey HSD post-hoc test. When the assumption of normality was not met, statistical comparisons were made using the non-parametric Kruskal–Wallis test, followed by Dunn’s multiple comparison. Analyses were performed using GraphPad Prism 8.0.1 (GraphPad Software, Boston, MA USA). A P-value < 0.05 was considered statistically significant.

## Results

### Part 1. Establishment of the endometriosis model in CD1 mice

#### Induction of endometriosis in CD1 mice

**Fig. 1:**
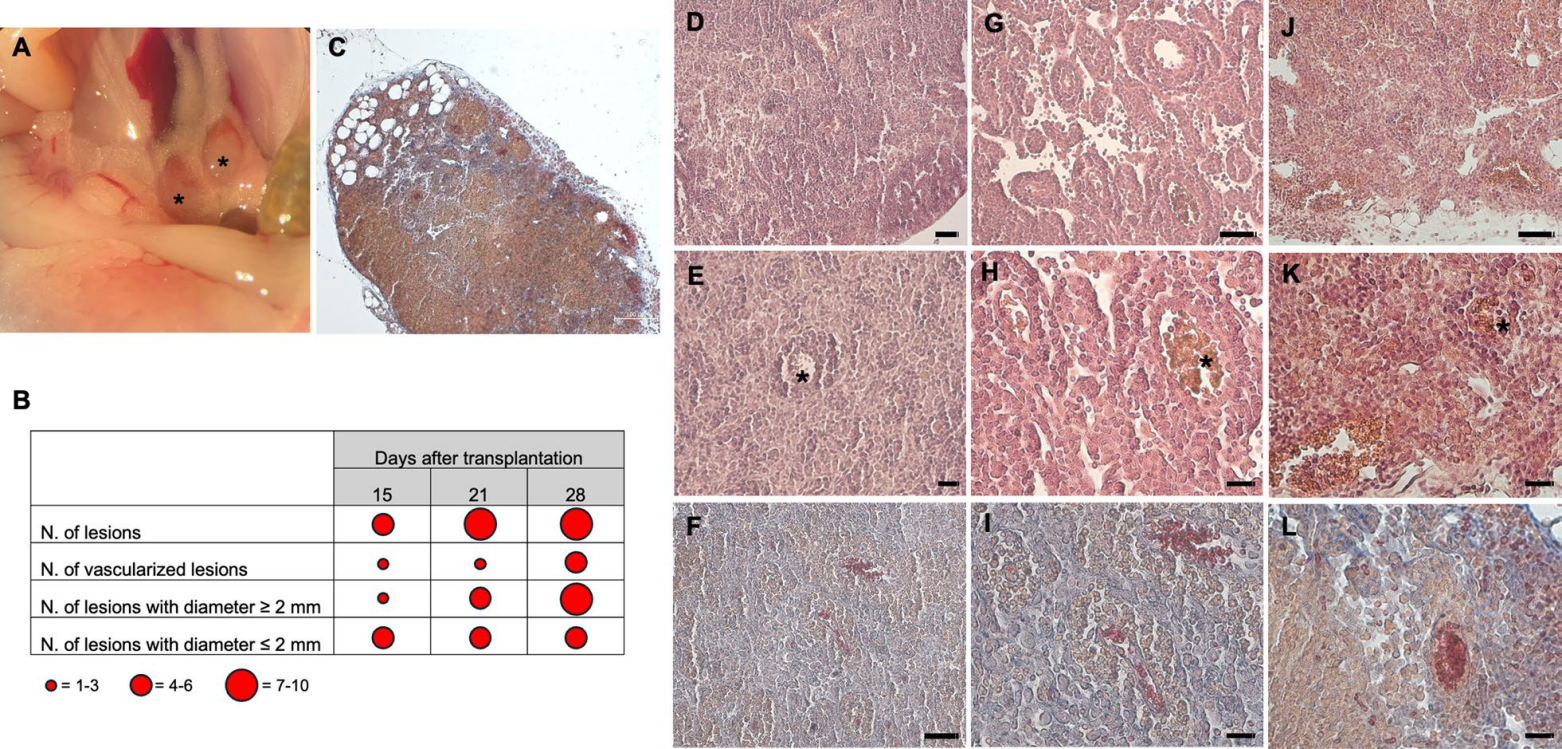
**A** Representative images of lesions isolated from the abdominal cavity of mice. Asterisks indicate lesions. **B** Mean number of lesions, vascularization and size at 15, 21 and 28 after the induction of endometriosis. Five-six mice per experimental group were employed. **C** H&E of a representative endometriotic lesion showing the external connective capsule. LM, mag: 5X. **D**–**L** H&E (**D**, **E**, **G**, **H**, **J**, **K**) and Trichrome AZAN (**F**, **I**, **L**) stainings of a representative endometriotic lesions from 15-days p.t. (**D**–**F**), 21 days p.t. (**G**–**I**) and 28 days p.t. (**J**–**L**) mice by LM. Asterisks indicate endometriotic foci: **D**, **G**, **J** LM, mag. 20X; bar: 50 µm. **E**, **F**, **H**, **I**, **K**, **L** LM, mag. 40X; bar: 20 µm

An endometriosis model was established in CD1 mice as previously described in other strains (Dabrosin et al. 2002; Xu et al. 2012). Although observations in endometriosis model induced in other mouse strains are usually performed 21 days p.t. (Burns et al. 2021), we decided to determine the optimal end point for our study in CD1 mice by sacrificing mice with ectopic endometrial grafts at 15, 21, and 28 days p.t. After each time point, mice were sacrificed and the presence of endometriotic lesions in the abdominal cavities was visually inspected. Lesions were present in the visceral peritoneum, intestines, and abdominal adipose tissue. A visual analysis of the lesions under the dissecting microscope was used to evaluate their number, size, and vascularization. Endometriotic lesions were present at all time points (Fig. 1A–C).

The number of lesions recovered in mice at 21 and 28 days p.t. was significantly higher than that observed in mice sacrificed at 15 days p.t. In addition, the number of lesions with a diameter ≤ 2 mm was the same in the three experimental groups, but the number of lesions with a diameter ≥ 2 mm was significantly higher in the groups sacrificed at the later time points compared to those observed in mice sacrificed at 15 p.t. Finally, the number of vascularized lesions observed in mice sacrificed at 28 days p.t. was significantly increased compared to the previous time points. The data are summarized in Fig. 1B.

#### Histological analysis of the lesions at 15, 21 and 28 days after endometriosis induction

The endometrium is the innermost layer of the uterine wall and rests on the underlying muscular layer (myometrium); it consists of a single layer of columnar epithelium with ciliated cells interspersed with mucus-secreting goblet cells and the lamina propria or stroma, which consists of loose connective tissue poor in fiber and rich in vessels and tubular glands producing a secretion rich in glycoproteins and glycogen. This upper highly vascularized functional layer is lined by the lower basalis, which does not undergo progesterone-mediated shedding, as the upper.

Histological analysis by H&E of endometriotic lesions allowed to highlight common structures in all isolated lesions 15, 21 and 28 days after induction of endometriosis. All lesions had a well-represented external connective capsule (Fig. 1C). Internally, the lesions were formed by endometrial tissue containing endometriotic foci which, as expected, had a homogeneous cellularity. Endometriotic foci were peculiar roundish structures with an internal presence of blood and/or flaky endometrial cells. The presence of blood within the foci was a distinctive feature that differentiates the foci from other tubular endometrial glands (Fig. 1E, H, K). The foci were generally lined by a simple cuboid epithelium, sometimes undergoing hyperplasia.

The presence of an endometrial pattern of both stromal and epithelial type was revealed, with the presence of some cystic structures.

The AZAN trichrome staining confirmed that endometriotic lesions showed encapsulation with an increasing number of endometrial foci containing blood (Fig. 1F, I, L).

#### Histological analysis of the ovaries at 15, 21 and 28 days after endometriosis induction

**Fig. 2:**
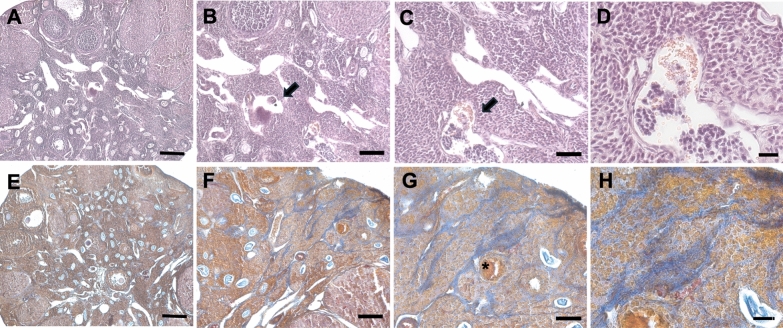
H&E (**A**–**D**) and Trichrome AZAN (**E**–**H**) stainings of endometriotic ovaries from 15 days p.t. mice by LM. **A**, **B**, **E**, **F** low magnification pictures of a representative endometriotic ovary. **C**, **D**, **G**, **H** high magnification showing the presence of endometriotic foci (arrows) and an encapsulated cyst (asterisk). **A**, **E** mag. 5X; bar: 200 µm; **B**, **F** mag. 10X; bar: 100 µm. **C**, **G** mag. 20X; bar: 50 µm. **D**, **H** mag. 40X; bar: 20 µm

LM observations of H&E stained ovarian sections revealed a time-dependent loosening of the compactness of the medulla due to the increased presence of endometriotic cysts, which appeared as encapsulated structures enclosing flaky endometrial cells. Endometriotic foci were identified as compact and basophilic cellular aggregates disseminated in the ovarian stroma, morphologically unrelated to specific elements of the ovary (Fig. 2A–D).

Trichrome AZAN staining revealed a time-dependent increase of fibrosis (not shown), as evidenced by the presence of a dense network of collagen fibers distributed in the septa of the medulla but especially, of the cortex (Fig. 2E, F).

### Part 2. Effects of D-chiro-inositol in the endometriosis model established in CD1 mice

#### Effect of D-chiro inositol and/or Dienogest administration on number, size, proliferation and vascularization of the lesions after D-chiro inositol and/or Dienogest administration

Based on the observations of part 1, in the second part of the study we used the non-surgical protocol used to create an endometriosis model in CD1 mice, with the 28 days p.t. being the best timepoint to evaluate the progression of the pathology.

Twenty-four hours after transplantation of ectopic endometrial tissue, mice were randomly assigned to the experimental groups and received the treatments described in the Materials and Methods section. During treatment, none of the mice showed signs of distress or other behavioral abnormalities.

At 28 days p.t., mice were sacrificed and visually inspected for the presence of endometriotic lesions. Lesions were present in the visceral peritoneum, intestines, and abdominal adipose tissue. Endometriotic lesions were found in all experimental groups (Table 1).

**Table 1:** Mean number of lesions, vascularization and size from mice of each group (mean ± SD)

	Lesion number	OneWay ANOVA p < 0.0001; ^§^p: *vs EMS; ^#^vs. DCI	Vascularized lesions	Kruskal Wallis p < 0.0001; ^¶^p: *vs EMS; ^#^vs. DCI	Lesion diameter ≥ 2 mm	Kruskal Wallis p = 0.0001; ^¶^p: *vs EMS; ^#^vs. DCI	Lesion diameter ≤ 2 mm	Kruskal Wallis p = 0.2368; -
EMS	8.71 ± 1.11	- -	7.00 ± 1.12	- -	5.29 ± 1.38	- -	3.43 ± 1.51	- -
DCI	3.86 ± 0.69	< 0.0001*; -	0.43 ± 0.55	< 0.0001*; -	1.43 ± 0.54	0.0001*; **-**	2.43 ± 0.79	- -
DCI + DG	5.29 ± 0.76	< 0.0001*; **0.0205**^**#**^	3.58 ± 0.79	0.5266*; **0.0257**^**#**^	3.57 ± 0.98	0.9870*; **0.0265**^**#**^	2.00 ± 1.16	- -
DG	4.71 ± 0.76	< 0.0001*; 0.2557^#^	1.57 ± 0.98	0.0043*; 0.5652^#^	2.29 ± 0.76	0.0140* > 0.9999#	2.43 ± 1.27	- -

Bold indicates statistical significance

^§^Tukey’s multiple comparison

^¶^Dunn’s multiple comparison

-: not analysed

**Fig. 3:**
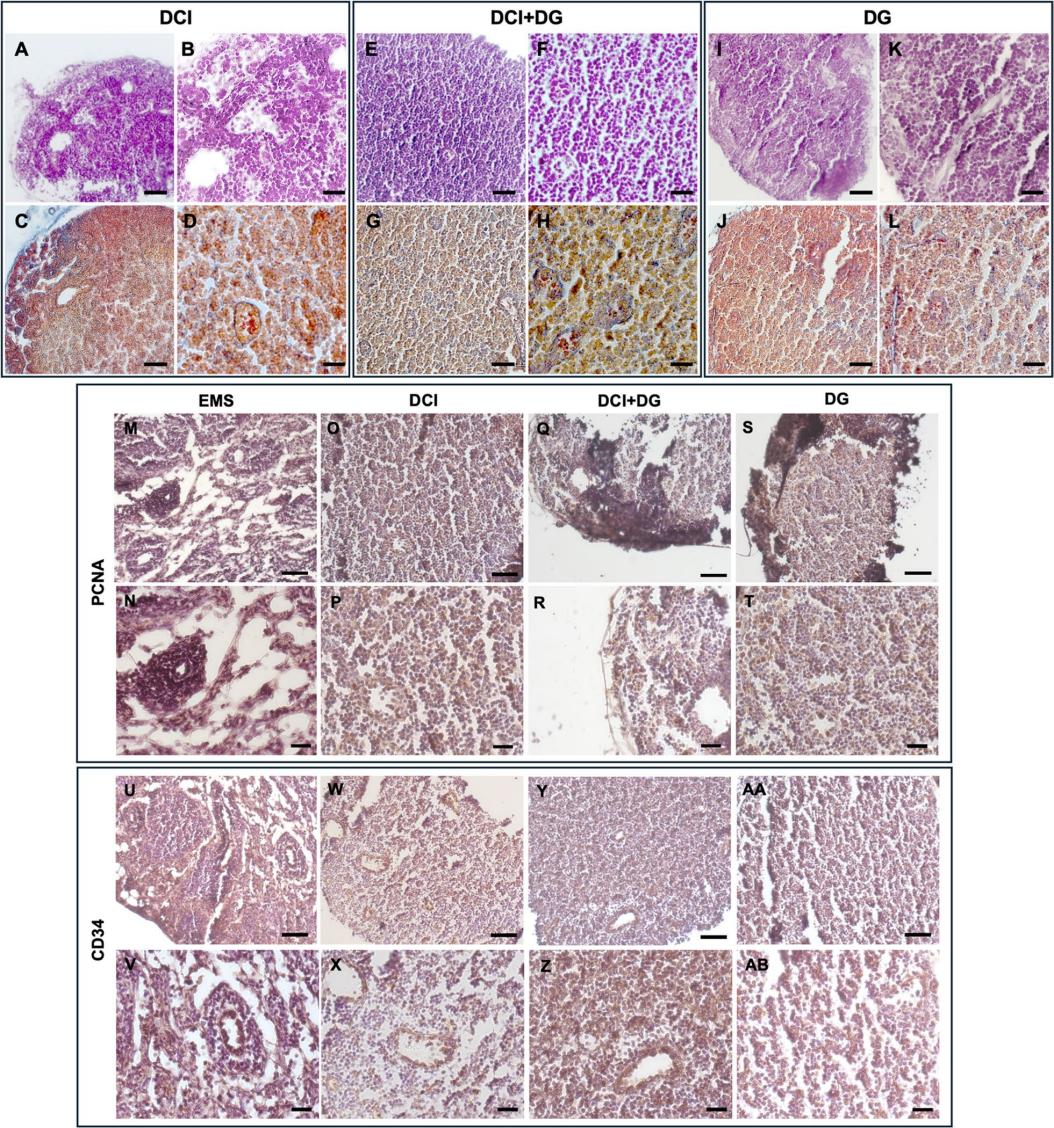
H&E (**A**–**B**) and Trichrome AZAN (**C**–**D**) stainings of representative endometriotic lesions in DCI-treated mice 28 days p.t. by LM. **A**, **D** mag. 10X; bar: 100 µm. **B**, **E** mag. 20X; bar: 50 µm. **C**, **F** mag. 40X; bar: 20 µm (**E**–**F**) and Trichrome AZAN (**G**, **H**) stainings of representative endometriotic lesions in DG + DCI-treated mice 28 days p.t. by LM. **E**, **G** mag. 20X; bar: 50 µm. **F**, **H** mag. 40X; bar: 20 µm. H&E (**I**–**K**) and Trichrome AZAN (**J**–**L**) stainings of representative endometriotic lesions in DG-treated mice 28 days p.t. by LM. **I**, **J** mag. 20X; bar: 50 µm. **K**, **L** mag. 40X; bar: 20 µm. **M**–**T** PCNA immunoreactivity in endometriotic lesions 28 days p.t. of control EMS (**M**, **N**), DCI-treated (**O**, **P**), DG + DCI (**Q**, **R**) and DG-treated (**S**, **T**)-treated mice by LM. **M**, **O**, **Q**, **S** LM, mag. 20X; bar: 50 µm. **N**, **P**, **R**, **T** mag. 40X; bar: 20 µm. **U**–**AB** CD34 immunoreactivity in endometriotic lesions 28 days p.t. of control EMS (**U**, **V**), DCI-treated (**W**, **X**), DG + DCI (**Y**, **Z**) and DG-treated (**AA**, **AB**)-treated mice by LM. **U**, **W**, **Y**, **AA** mag. 20X; bar: 50 µm. **V**, **X**, **Z**, **AB** mag. 40X; bar: 20 µm

As expected, mice of the EMS control group had a significantly higher number of lesions compared to the other treatments. In addition, the DCI group was significantly more effective than the DCI + DG group in reducing the number of lesions. In mice receiving DCI and/or DG after endometriosis induction, lesion analysis showed a reduction in the size of the endometrial foci after H&E staining. The lesions were characterized by cytogenic stroma with small and uniform cells, with rounded nuclei and very little cytoplasm, within which foci showed a typical appearance characterized by monostratified epithelium (Fig. 3A–L). In addition, AZAN trichrome staining revealed a reduction in endometrial foci containing blood (Fig. 3A–L.).

When we focused on the size of the lesions, we found that about two-thirds of the lesions had a diameter ≥ 2 mm, and one-third had a size ≤ 2 mm. Endometriotic foci and the stromal component of lesions in the EMS group were characterized by the nuclear presence of PCNA, a marker of proliferation. Our data indicated that mice receiving DCI or DG after induction of endometriosis had a reduced number of lesions ≥ 2 mm. The administration of DCI + DG did not influence lesion size compared to the EMS group, although a slight non-significant reduction was observed. Consistent with these observations, a reduction in PCNA-positive cells was observed in lesions from mice treated with DCI and/or DG. This PCNA reduction was more pronounced in the DCI group (Fig. 3M–T).

As expected from the preliminary results, most of the lesions isolated in the control group were vascularized. This finding was further supported by the presence of the transmembrane protein CD34 in EMS lesions, showing the expression of the marker both in the epithelial cells of the endometriotic foci and in the stroma. A low level of vascularization was found in all treated mice compared to the EMS group. In particular, DCI and DG had similar effects, and each individually had a greater effect than the combination of the two. Consistent with these results, immunohistochemical analysis of CD34 revealed a clear presence of the protein in the EMS group, while the treatment with DCI significantly reduced the presence of this marker (Fig. 3U–AB).

#### Effects of D-chiro inositol and/or Dienogest administration on serum estradiol level and ovarian aromatase

**Fig. 4:**
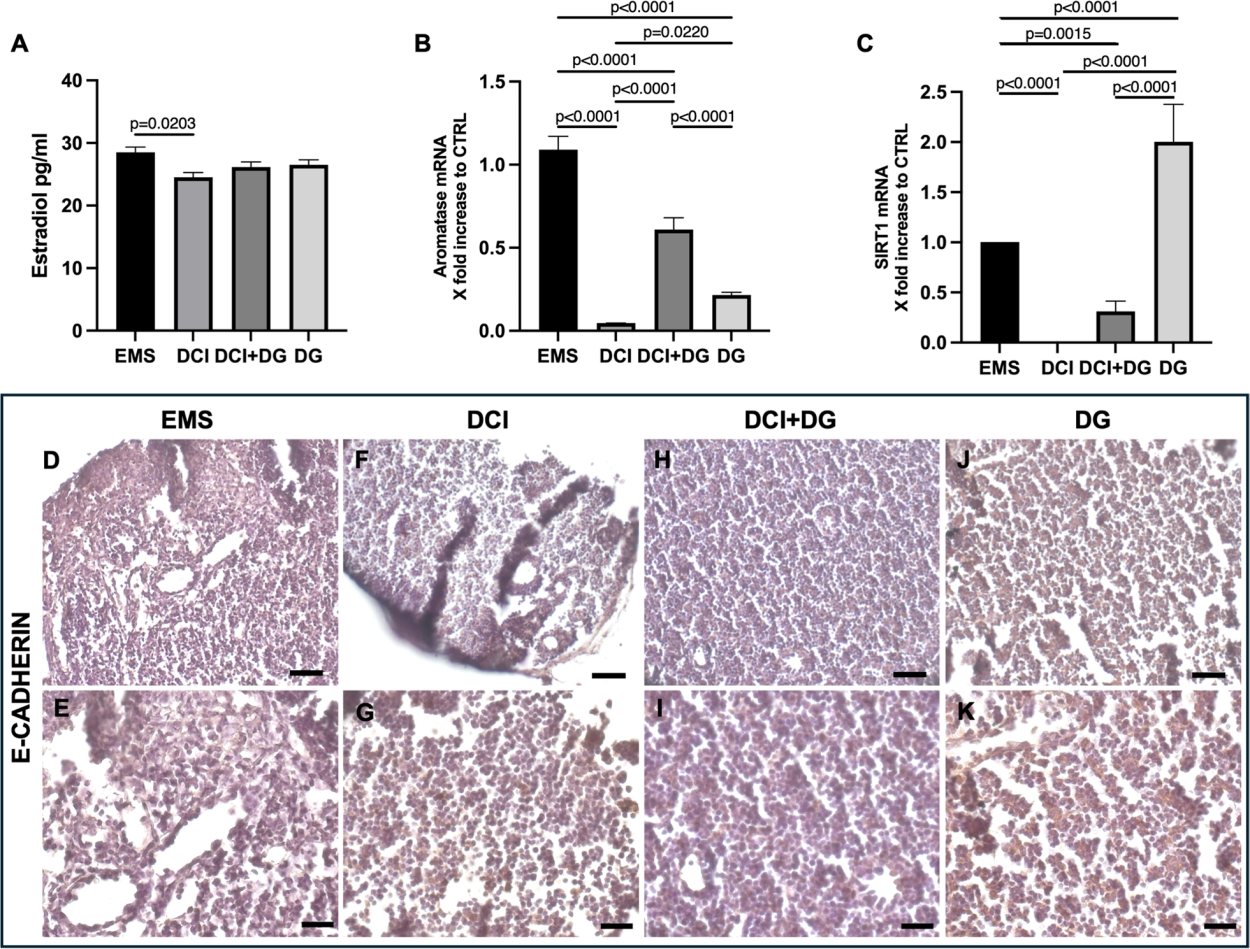
**A** Serum estradiol levels in the different experimental classes. Three mice per experimental group were employed. Experiments were done in triplicate. One-way ANOVA: p = 0.0322, followed by Tukey HSD multiple comparisons. **B** Aromatase gene expression in ovaries. Three mice per experimental group were employed. Experiments were done in triplicate. One-way ANOVA: p < 0.0001, followed by Tukey HSD multiple comparisons. **C** SIRT1 gene expression in lesions. Three mice per experimental group were employed. Experiments were done in triplicate. One-way ANOVA p < 0.0001, followed by Tukey HSD multiple comparisons. **D**–**K** E-Cadherin immunoreactivity in endometriotic lesions 28 days p.t. of control EMS (**D**, **E**), DCI-treated (**F**, **G**), DG + DCI (**H**, **I**) and DG-treated (**J**, **K**)-treated mice by LM. **D**, **F**, **H**, **J** mag. 20X; bar: 50 µm. **E**, **G**, **I**, **K** LM, mag. 40X; bar: 20 µm

To monitor hormonal influences and evaluate treatment efficacy, we measured and compared the circulating estradiol level in different experimental groups. As shown in Fig. 4A, mice receiving DCI after endometriosis induction had a significant decrease in estradiol levels compared to the EMS group. Treatment with DCI + DG or DG was not effective in reducing the circulating estradiol.

Since aromatase is the ovarian enzyme that converts androgens to estrogens and affects estradiol levels, we then focused on its expression in the ovary. All treatments reduced aromatase expression compared to the control group. Consistent with estradiol results, DCI mice showed a reduction in the aromatase transcript that was significantly different from the DG and DCI + DG groups. In addition, the DG reduction was more pronounced than that induced by DCI + DG. Finally, we investigated whether lesions expressed aromatase to sustain their proliferation, but the signal was not detectable (Fig. 4B).

#### Effects of D-chiro inositol and/or Dienogest administration on epithelial-mesenchymal transition (EMT) of endometriotic lesions

EMT is a key process that contributes to the pathogenesis of endometriosis. Here, we focused on the expression of Sirt1, which modulates the genes involved in EMT and potentially promotes the transition of epithelial cells to mesenchymal-like cells in endometrial tissue. Our results showed that Sirt1 gene expression was not detectable in the lesions of mice receiving DCI. In the DCI + DG group the level of Sirt1 expression was lower in comparison to the control and DG group. Lesions from mice receiving DG showed a higher level of Sirt1, which was twice that observed in the EMS group (Fig. 4C).

During EMT, there is a loss of E-cadherin expression, which facilitates the dissociation of cells from their epithelial structure, allowing increased migration and invasion characteristic of endometriosis. Consistent with these observations, immunohistochemical analysis of E-cadherin highlighted reduced staining in the EMS group compared to the treated mice (Fig. 4D–K). In all treatments, the E-cadherin staining was higher than the control, although it is not clearly visible in DCI due to the flakiness of the tissue (Fig. 4F, G).

#### Effects of D-chiro inositol and/or Dienogest administration on morphological and functional characteristics of ovaries from endometriotic mice

**Fig. 5:**
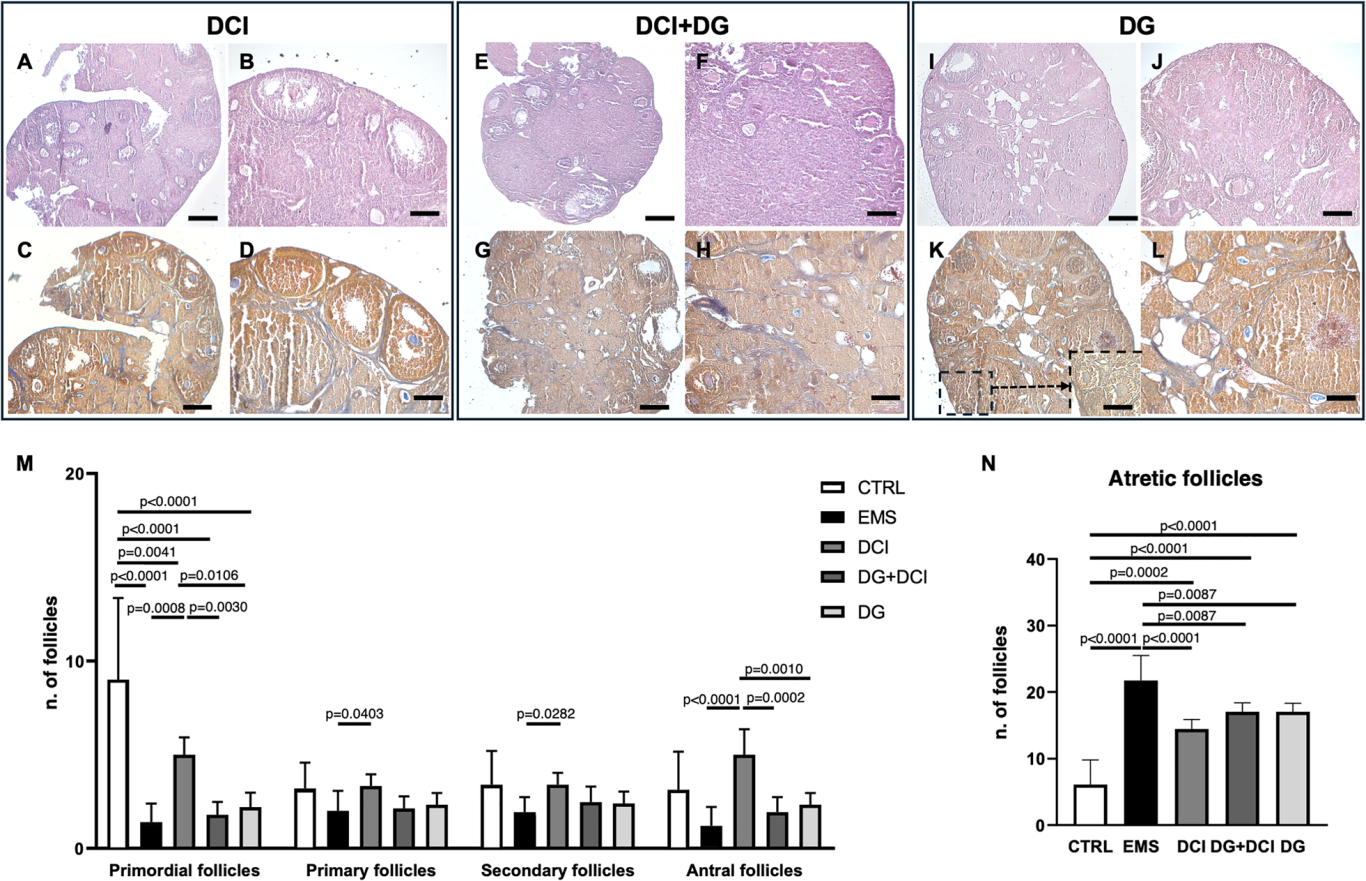
H&E (**A**–**B**) and Trichrome AZAN (**C**–**D**) stainings of a representative endometriotic ovary 28 days p.t. from DCI-treated mice by LM. **A**, **C** mag. 5X; bar: 200 µm; **B**, **D** mag. 10X; bar: 100 µm. H&E (**E**, **F**) and Trichrome AZAN (**G**, **H**) stainings of a representative endometriotic ovary 28 days p.t. from DG + DCI-treated mice by LM. **E**, **G** mag. 5X; bar: 200 µm; **F**, **H** mag. 10X; bar: 100 µm. H&E (**I**, **J**) and Trichrome AZAN (**K**, **L**) stainings of a representative endometriotic ovary 28 days p.t. from DG-treated mice by LM. **I**, **K** mag. 5X; bar: 200 µm; **J**, **L** mag. 10X; bar: 100 µm. **M** Ovarian follicle count. Values are expressed as mean ± SD. Differences were evaluated via ANOVA (p < 0.05) followed by Tukey HSD post-hoc test: * p < 0.05; ** p < 0.01. **N** Total atretic follicles. Values are expressed as mean ± SD. Differences were evaluated via ANOVA (p < 0.05) followed by Tukey HSD post-hoc test: * p < 0.05; ** p < 0.01

Observation of ovarian sections by LM showed that all treatments induced a marked morphological improvement of the organ in terms of compactness, reduction of endometriotic lesions and increased number of growing ovarian follicles. In particular, mice from DCI group showed a very compact ovary with reduced vascularization in the medulla and fibrosis. In contrast, the DCI + DG group showed an ovary with reduced fibrosis and follicular atresia. In the DG group, there was a more compact ovary with a marked reduction in atresia and fibrosis and a greater vascularization in the medulla compared to the EMS group (Fig. 5A–L).

**Fig. 6:**
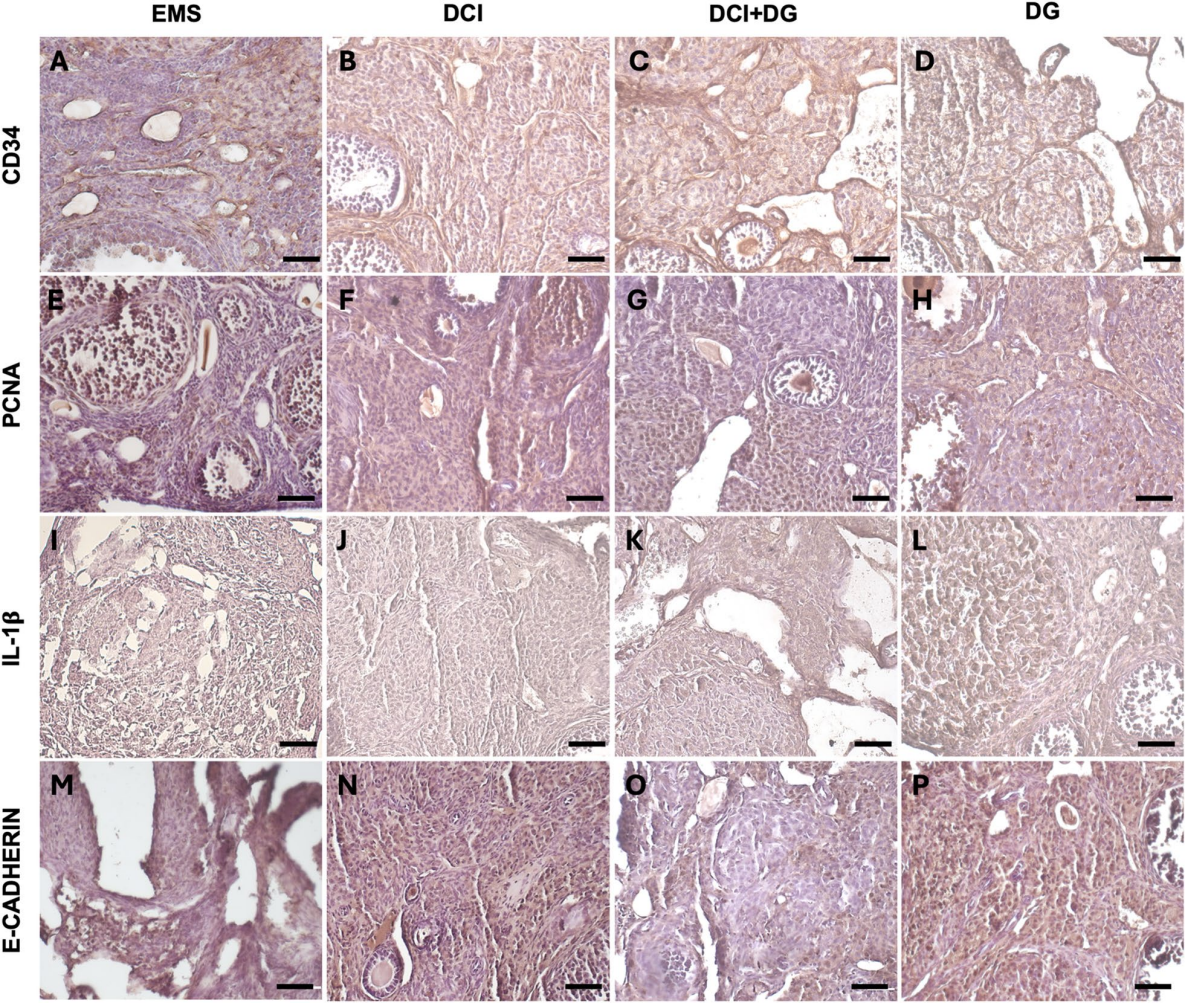
**A**–**D** CD34 immunoreactivity in endometriotic ovary 28 days p.t. of control EMS (**A**), DCI (**B**), DG + DCI (**C**) and DG (**D**)-treated mice by LM. mag. 20X; bar: 50 µm; **E**–**H**) PCNA immunoreactivity in endometriotic ovary 28 days p.t. of control EMS (**E**), DCI (**F**), DG + DCI (**G**) and DG (**H**)-treated mice by LM. mag. 20X; bar: 50 µm. **I**–**L** IL-1β immunoreactivity in endometriotic ovary 28 days p.t. of control EMS (**I**), DCI (**J**), DG + DCI (**K**) and DG-treated (**L**)-treated mice by LM. mag. 20X; bar: 50 µm. **M**–**P** E-Cadherin immunoreactivity in endometriotic ovary 28 days p.t. of control EMS (**M**), DCI (**N**), DG + DCI (**O**) and DG (**P**)-treated mice by LM. mag. 20X; bar: 50 µm

In accordance with histological observations, immunohistochemical evaluation of CD34, which is known to be expressed in endothelial cells of blood vessels during vascularization processes, revealed the presence of CD34-positive staining in the cells of the cystic structures and in the stroma of the endometriotic ovary. By contrast, a significant reduction in CD34 was observed with DCI treatment, moderate with DG and mild with the combined DG + DCI treatment (Fig. 6A–D).

To monitor cell proliferation and identify changes associated with ectopic endometrial growth, we relied on immunohistochemical analysis of PCNA expression. Our results showed a reduction in PCNA-positive cells in the ovaries of mice receiving DG or DCI, with a more pronounced effect in the latter. By contrast, high levels of PCNA staining were observed in ovaries from DG + DCI treatment (Fig. 6E–H).

Since the inflammatory process is very active in endometriosis, we focused on IL-1β positive staining to evaluate possible changes induced by treatments based on DCI and/or DG. As expected, the pro-inflammatory cytokine IL-1β was highly abundant in the EMS group. The treatment with DCI induced a marked reduction of IL-1β positive staining compared to the EMS group. A mild effect was also observed in the ovaries of DCI + DG and DG mice (Fig. 6I–L).

Finally, we evaluated the expression level of E-Cadherin, which plays an important role in normal follicle development and function (Piprek et al. 2020). Immunohistochemical analysis revealed a very low level of E-Cadherin staining in the EMS group. By contrast, higher expression of E-Cadherin was detected in the ovaries of mice receiving DCI and/or DG after endometriosis induction. In particular, higher expression of E-Cadherin was observed in the ovaries of the DCI and DG groups compared to the ovaries of mice receiving the combined DG + DCI treatment (Fig. 6M–P).

#### Effects of D-chiro inositol and/or Dienogest administration on ovarian reserve

To investigate the effects of endometriosis on ovarian reserve and follicle growth, we compared the number of follicles observed in ovarian sections of EMS mice with mice receiving different treatments after endometriosis induction and used a further control group represented by mice without endometriosis. As shown in Fig. 5M, induction of endometriosis altered ovarian reserve, as demonstrated by a reduced number of primordial follicles. Administration of DCI was the only treatment able to preserve the population of primordial follicles. Ovaries from DG and DCI + DG groups presented a reduction in ovarian reserve similar to that of the EMS control group. Although induction of endometriosis did not affect the number of primary, secondary, and antral follicles in comparison to untreated mice, DCI increased the population of primary to antral follicles. In addition,, endometriosis induced an increase in atretic follicles compared to untreated mice. All treatments similarly reduced the numbers of atretic follicles, although none restored follicle atresia to levels similar to untreated mice (Fig. 5N).

## Discussion

In the present study, we investigated the potential therapeutic effect of DCI in the treatment of endometriosis. DCI appears promising as a treatment for estrogen-dependent diseases like endometriosis, given its natural origin and lack of side effects typical of hormonal therapies or enzyme inhibitors like letrozole. Clinical evidence supports its use in conditions such as endometrial hyperplasia, with oral formulations demonstrating good bioavailability and safety (Porcaro et al. 2023).

Mouse models of endometriosis allow controlled studies of disease mechanisms, including adhesion and immune responses, and allow testing of potential treatments at the molecular, cellular and histological levels. Although endometriosis is not observed in rodents, it can be induced by surgical and non-surgical methods with the development of lesions similar to those observed in human pathology (Dorning et al. 2021). In the present work, we developed a non-surgical model of endometriosis in CD1 mice. Based on a method mainly applied on BALB/C mouse strain (Pittaluga et al. 2010; Yan et al. 2019; Woo et al. 2020; Burns et al. 2021), we validated for the first time the induction of endometriosis through intraperitoneal injection of endometrial fragments in CD1 mice and observed a time-dependent development of endometriotic lesions. Our results show that: (i) the non-surgical protocol used here represents an effective method to generate an endometriosis model in CD1 mice, with no signs of rejection; and (ii) 28 days p.t. was, in our hands, an optimal and reasonable time point to evaluate the progression of the pathology, considering the parameters of lesion number, growth and vascularization.

Using this model, the efficacy of daily administration of 0.4 mg DCI was evaluated by treating mice for 28 days. We measured circulating estradiol levels and expression of aromatase, the enzyme responsible for estradiol production and analyzed histological and molecular changes in endometriotic lesions and ovaries, focusing on proliferation, vascularization, EMT and invasion. As a positive control, we used DG, a progestin commonly used in the treatment of endometriosis symptoms (Vercellini et al. 2009). A combination of DCI and DG, with half the dose of each compared to single compounds, was also tested to investigate a possible synergistic effect.

All treatments employed in the present study showed a reduction in the number of lesions, their size and relative vascularization compared to the EMS group, along with reduced estrogen production and aromatase expression. Among all, administration of DCI after endometriosis induction was proved to be the most effective treatment. These results are consistent with the hypothesis by Carvalho and colleagues regarding the use of aromatase inhibitors in the treatment of this disorder (Carvalho et al. 2011). In fact, extrauterine endometrial tissue is a source of estrogens and these stimulate the synthesis of prostaglandin E2 (PGE2), a potent inducer of aromatase activity in the endometrium. This mechanism appears to underlie of the vicious cycle that leads to the growth of new ectopic endometrial tissue (Carvalho et al. 2011; Słopień and Męczekalski 2016; Mori et al. 2018). The high expression of PGE2 in the endometriotic lesions leads to the establishment of a positive feedback loop between the steroidogenic factor-1 (SF1), the increase in aromatase activity and the vascular endothelial growth factor (VEGF) (Bulun and Simpson 2008). Thus, the results obtained with DCI treatment may be due to its inhibitory action on aromatase, the enzyme capable of perpetuating this vicious cycle (Laganà et al. 2018; Monastra et al. 2021; Gambioli et al. 2021a). Indeed, our study showed that aromatase expression was reduced in the ovaries of mice receiving DCI and/or DG compared to the control group, but the reduction by DCI was the most pronounced, and DCI was the only treatment able to reduce plasma levels of estradiol. Aromatase inhibition may prevent abnormal production of estradiol that promotes lesion growth, supporting the hypothesis that reduced vascularization of endometriotic lesions and their reduction in number may be due to aromatase inhibition by DCI. Although previous studies have reported aromatase expression in endometriotic tissue and lesions (Da Costa et al. 2022), its level was not detectable in lesions isolated from EMS or treated mice. Further study may help to clarify whether DCI modulates pathways beyond those mediated by aromatase inhibition. In addition, DCI may influence lesion growth by acting on PI3K signalling. Indeed, aromatase is known to promote PI3K, with consequent activation of key pathways that maintain cell survival and proliferation (Amaral et al. 2020). Aromatase inhibitors, such as letrozole, decrease PI3K activation and affect downstream effectors such as AKT, mTOR, and p53, resulting in a shift toward increased apoptosis and reduced cellular viability (Hoeflich et al. 2016). Further studies focusing on pro- and anti-apoptotic factors may provide critical insights into how reduced aromatase activity influences lesion regression and therapeutic efficacy.

Epithelial-mesenchymal transition (EMT) is a biological process in which epithelial cells lose their characteristics and acquire migratory and invasive properties typical of mesenchymal cells (Akhmetkaliyev et al. 2023). Recently, EMT has emerged as key mechanism contributing to the pathogenesis of endometriosis (Chen et al. 2020). SIRT1 is a NAD^+^-dependent deacetylase that plays a crucial role in cellular stress responses, metabolism, and inflammation (Tatone et al. 2018) and is involved in the transition of epithelial cells into mesenchymal-like cells in endometrial tissue (Wang et al. 2022). It is known that SIRT1 expression is higher in endometriotic lesions compared to eutopic endometrium (Kim et al. 2021). Furthermore, in human endometriotic lesions, SIRT1 overexpression has been demonstrated to promote the EMT process and senescence escape (Wang et al. 2022), as occurs in most tumors (Ansieau et al. 2008), supporting the hypothesis that senescence escape of endometrial epithelial cells could contribute to the acquisition of metastatic characteristics observed in endometriosis (Wang et al. 2022). Interestingly, our study showed that all treatments decreased Sirt1 gene expression, which reaches undetectable levels in lesions isolated from DCI. Although the mechanisms by which DCI modulates SIRT1 availability remain to be elucidated, an indirect effect of DCI on SIRT1 may be related to the modulation of insulin signaling (Aventaggiato et al. 2022; Shi et al. 2023). In contrast to SIRT1, E-cadherin appears to play opposed role in the regulation of EMT in endometriosis (Wang et al. 2022). E-cadherin is a calcium-dependent adhesion molecule that is crucial for maintaining epithelial cell integrity. During EMT, loss of E-cadherin expression facilitates the dissociation of cells from their epithelial structure, allowing for endometrial tissue remodeling, increased migration and invasion characteristic of endometriosis (Zhu et al. 2018; Wang et al. 2022). Furthermore, E-cadherin interacts with various signaling pathways (e.g., Wnt/β-catenin) that are involved in EMT processes (Zhu et al. 2018; Wang et al. 2022). Consistent with these studies, our data demonstrated that treatments with DCI and/or DG effectively reduced the progression of EMT, with DCI being more effective than DG or the combination of DG with a low dose of DCI. In this context, we can hypothesize that the improved efficacy of DCI treatment on the development of endometriosis compared to DG may be related to the inhibition of the EMT process, as already described in other systems (Monti et al. 2023).

Endometriosis can affect ovarian function in a variety of ways (Gambigliani Zoccoli and La Marca 2024; Park et al. 2024). Local chronic inflammation and fibrotic alteration of the ovarian cortex may be one of the causes of reduced ovarian function (Park et al. 2024). Our study effectively demonstrates that DCI can induce favorable morphological changes in ovaries affected by endometriosis, with distinct differences in their effects on vascularization, inflammation, and ovarian reserve. Endometriosis is characterized by chronic inflammation with immune dysregulation and release of pro-inflammatory cytokines which can have negative effects on the ovaries (Ahn et al. 2015; Boucher et al. 2022). Recent studies have demonstrated the activation of the inflammasome in the ovarian microenvironment of IVF patients with endometriosis, as evidenced by increased caspase-1 and elevated levels of IL-1β and IL-18 in follicular fluid (Fonseca et al. 2023). In accordance with these observations, we found high levels of IL-1β in the endometriotic lesions of control mice, which were reduced by administration of DCI or DG. These anti-inflammatory effects of DCI are not surprising, as a plethora of studies have described its anti-inflammatory effects in other organs and systems (Fortis-Barrera et al. 2013; Zhang et al. 2018; Yang et al. 2022). As a possible mechanism underlying this effect, it can be considered that DCI enhances insulin activity, which can potentially reduce inflammation as it occurs in systemic metabolic syndromes (Owczarczyk-Saczonek et al. 2018; Gambioli et al. 2021b; Menichini et al. 2022).

Our analysis revealed that all treatments increased the expression of ovarian E-cadherin, which was negatively affected by endometriosis in our mouse model. E-cadherin is known to regulate ovarian epithelial homeostasis by regulating cell–cell adhesion. By playing a relevant role in normal follicle development and function, it reduces the establishment of inflammatory processes in the ovarian microenvironment in endometriotic mice (Piprek et al. 2020). A decreased ovarian reserve is the main deterioration of ovarian function in women with endometriosis (Kitajima et al. 2011). Reduced availability of competent follicles, together with an inflammatory environment of intrafollicular environment, may result in the production of less competent oocytes and impaired production of ovarian steroid hormones. In this context, it is of considerable interest that the ovaries of mice receiving DCI after endometriosis induction have a significantly higher number of primordial follicles compared to other treatment groups. Consistent with observations in patients with endometriosis (Tian et al. 2021), we have observed a trend toward reduced antral follicle number in endometriotic mice that was prevented by DCI treatment.

In conclusion, this work has allowed us to validate an easily reproducible model of endometriosis, that is less invasive than the traditional surgical method. Thanks to the spontaneous adhesion of the endometrial tissue, this model reproduces the natural process that leads to the onset and progression of the pathology including the effects on the ovary in a 28-day window. The use of this model has allowed us to discover the beneficial effects of DCI showing promising results in the treatment of endometriosis and paving the way to future investigations aimed at introducing this compound into clinical practice. Further research could explore the long-term effects of these treatments on fertility outcomes and the underlying mechanisms by which they improve ovarian function in endometriosis. In addition, investigating the effects of combination therapy on different pathways may provide broader insights into strategies for managing endometriosis-related ovarian dysfunction.

## Supplementary Information

Supplementary material 1.

Supplementary material 2.

## Data Availability

All data generated or analyzed during this study are included in this published article.

## Abbreviations

DCI: D-chiro inositol
DG: Dienogest
EMT: Epithelial mesenchymal transition
EMS: Endometriosis
IVF: In vitro fertilization
LM: Light microscopy
