# Supplementary material for: D-chiro-inositol effectively counteracts endometriosis in a mouse model
Source: Mol Med. 2025 Apr 11;31:134. doi: 10.1186/s10020-025-01178-6 (PMC11987403; doi:10.1186/s10020-025-01178-6)
Supplement: Supplementary file 1 — Supplementary material 1. [file 10020_2025_1178_MOESM1_ESM.docx]

**Supplementary Table 1.** ARRIVE Essential 10

| Study Design | Part 1: Establishment of the endometriosis (EMS) model in CD1 mice.  Groups: EMS 15, 21 or 28 days post transplantation (p.t.).  Part 2: Effects of D-chiro-inositol (DCI) in the EMS model established in CD1 mice  Groups: control, EMS, DCI (0.4 mg/day), DG (dienogest, 0.67 ng/day), DCI+DG (0.2 mg + 0.33 ng/day).  Experimental unit: single mouse. |
| --- | --- |
| Sample Size | Part 1: Total n. 20 mice: n. 4 donors; n. 5 EMS 15 days p.t.; n. 5 EMS 21 days p.t.; n. 6 EMS 28 days p.t.  Part 2: Total n. 38 mice: n. 7 donors; n. 7 EMS, n. 7 DCI (0.4 mg/day), n. 7 DG (0.67 ng/day), n. 7 DCI+DG (0.2 mg + 0.33 ng/day); n. 3 control.    Total number of mice: 58 units.  Sample size was determined a priori using G*Power3 software. |
| Inclusion and Exclusion Criteria | Inclusion criteria for donor mice: female mice at the diestrus stage of the ovulatory cycle.  Exclusion criteria were established a priori. They were defined as the occurrence of weight loss, abnormal behaviors, or other signs of acute stress, upon which the subject would have been removed from the study and the treatment immediately discontinued.  No animals were excluded from the study. |
| Randomisation | Randomized allocation of mice to treatment groups.  Confounders were not controlled. |
| Blinding | The day after EMS induction, one researcher randomly allocated mice in 8 cages; another researcher assigned the experimental treatment to the different cages.  At the time of sacrifice, the researcher inspecting the peritoneal cavity was unaware of the experimental group.  Histological analyses were conducted in a blinded manner. |
| Outcome Measures | Part 1: Establishment of the best timepoint after EMS induction by evaluating: lesion number, size, vascularization under light microscopy (LM), analysis of histological aspect (H&E, trichrome AZAN stain). Ovarian histological aspect (H&E, trichrome AZAN stain) was also analysed.  Results from part 1 were employed for the set up of part 2.  Part 2. Effects of D-chiro-inositol (DCI) in the EMS by evaluating:  lesion number, size (LM observation and PCNA immunolocalization), vascularization (LM observation and CD34 immunolocalization), EMT (E-CAD immunocalization and Sirt1 expression). It was also analysed ovarian histological aspect (H&E, trichrome AZAN stain), proliferation, vascularization and inflammation (PCNA, CD34 and IL-1beta, respectively), and function (aromatase expression, ovarian follicle count and E-CAD immunolocalization). Serum estradiol levels were also measured.  All lesions were counted and visually observed to establish size and the presence of vessels. Then, the lesions from each animal were processed for histo-immunological analysis or for testing gene expression.  One ovary from each mouse was destined to histo-immunological analysis, one ovary for gene expression.  Serum was collected from each mouse. Serum from 2-3 mice from the same experimental group was pooled prior to estradiol analysis. |
| Statistical Methods | Normal distribution was assessed by Shapiro-Wilk test. Samples with normal distribution, were compared by applying by One-way ANOVA, followed by Tukey HSD post-hoc tests. When the assumption of normality was not met, statistical comparisons were performed using the non-parametric Kruskal-Wallis test, followed by Dunn’s multiple comparison.  GraphPad Prism 8.0.1 software was used.  Data assumptions checked and significance set at p < 0.05. |
| Experimental Animals | Species: Outbred CD-1 mice, female, 4-6 weeks of age, 20-25 g.  Source: Charles River Laboratories. |
| Experimental Procedures | Donors: single intraperitoneal (i.p.) injection of 5 10 I.U. pregnant mare serum gonadotropin (PMSG) to induce folliculogenesis and endometrial cell proliferation 46 hours prior to sacrifice.  EMS induction via non-surgical intraperitoneal transplantation of endometrial fragments from the donors. Donor to recipient ratio was 1:4.    Part 1: At 15, 21 and 28 days after the induction of EMS, mice were sacrificed and lesions and ovaries were collected.  Part 2: The day after the induction of EMS, drinking water was supplemented for 28 days with:  - Nothing, EMS group;  - 0.4 mg/2 ml D-chiro-inositol, DCI group;  - 0.67 ng/2 ml Dienogest, DG group;  - 0.2 mg D-chiro-inositol and 0.33 ng DG, DCI+DG group.  28 days after the induction of EMS, all mice were sacrificed and lesions, serum and ovaries collected. |
| Results | In brief:  Part 1: EMS 28 p.t. is being the best timepoint to evaluate the progression of the pathology. Summary statistics reported in the text, including mean ± SD.  Part 2: DCI administration counteract EMS and is worth further studies. Summary statistics reported in the text, including mean ± SD.  For further details see the text. |
