# Supplementary material for: D-chiro-inositol effectively counteracts endometriosis in a mouse model
Source: Mol Med. 2025 Apr 11;31:134. doi: 10.1186/s10020-025-01178-6 (PMC11987403; doi:10.1186/s10020-025-01178-6)
Supplement: Supplementary file 2 — Supplementary material 2. [file 10020_2025_1178_MOESM2_ESM.docx]

**Supplementary Table 2.** ARRIVE Recommended Set.

| Abstract | Part 1: The goal of the first part of the study was to establish the best timepoint for the study of endometriosis in CD1 female mice. The analysis of lesion number, size and vascularization, in association with ovarian morphology, it emerged that 28 p.t. represented the best timepoint to evaluate the progression of the pathology.  Part 2: The main objective of the second part of the study was to evaluate the effects of DCI administration in counteracting the progression of endometriosis in CD1 female mice. DCI effects were compared with those induced by DG, the progestin medication commonly used for the treatment of endometriosis. We analyzed and compared lesion number, size and vascularization, histology, EMT markers, in association with changes in ovarian morphology, inflammation and function, and estradiol production in different experimental groups. Our data revealed that administration of DCI is a valuable approach worth further studies. |
| --- | --- |
| Background | Endometriosis is characterized by peritoneal inflammation, estrogen dependence, and angiogenesis, contributing to disease progression and resistance to progesterone. Current treatments focus on reducing estrogen levels using hormonal therapies such as GnRH agonists, oral contraceptives, and aromatase inhibitors, but these have limited efficacy and significant side effects. Given the urgent need for alternative therapies, D-chiro-inositol (DCI) has emerged as a potential candidate due to its ability to downregulate aromatase expression, thereby reducing estrogen production. Previous studies have demonstrated its effectiveness in restoring ovarian function in PCOS models, supporting its potential use in estrogen-dependent conditions like endometriosis. Unlike hormonal treatments, DCI has a favorable safety profile and bioavailability, making it a promising therapeutic option.  The murine model is essential as endometriosis occurs only in primates, and validated 3D human cell cultures are lacking. It enables studying pathogenetic mechanisms and therapies in a technically simple, low-stress system already validated for endometriosis research. |
| Objectives | Part 1: The goal of the first part of the study was to establish the best timepoint for the study of endometriosis in CD1 mice.  Part 2: The main objective of the second part of the study was to evaluate whether DCI administration counteracts the progression of endometriosis in CD1 mice and to compare DCI effects with DG, the progestin medication for commonly used for the treatment of endometriosis. |
| Ethical Statement | Approved by the internal ethics committee and the Italian Ministry of Health (authorization no. 917/2023-PR). |
| Housing and Husbandry | Temperature-controlled housing, 12h light/dark cycle, ad libitum food and water. Presence of environmental enrichment. |
| Animal Care and Monitoring | Mice were daily checked for signs of their health status. No animals showed signs of pain, stress or discomfort. No unexpected adverse events reported. |
| Interpretation/Scientific Implications | Part 1. The best timepoint for the study of endometriosis in CD1 mice is 28 days p.t..  Part 2. Treatment with DCI, alone or with DG, significantly reduced the number, size, and blood supply of endometriotic lesions. Notably, lesions in the DCI group showed reduced vascularization. Histological analysis confirmed fewer and smaller lesions across all treatment groups, with the strongest effects in the DCI group. DCI also reduced factors associated with lesion growth, cell proliferation, and new blood vessel formation, while improving ovarian function by lowering estrogen production and increasing the number of early-stage follicles. These findings suggest that DCI counteracts the progression of endometriosis lesion and support ovarian health, offering a potential non-hormonal therapeutic approach for endometriosis.  The main limitation of this study is that the treatment with DCI was administered for a short period, and its long-term effects remain unknown. |
| **Generalisability/Translation** | The study was conducted in an animal model, the findings may not be directly translatable to humans. Further research is needed to assess the long-term impact of DCI and to confirm its potential therapeutic applications in clinical settings. |
| **Protocol Registration** | Protocol prepared before the study, then it was submitted to and approved by the internal ethics committee and Italian Ministry of Health (authorization no. 917/2023-PR). |
| **Data Access** | Data available upon request. |
| **Declaration of Interests** | No competing interests.  Funding sources and roles acknowledged in the text. |
